# Does Test Duration Matter? Comparing 1‐s and 5‐s Isometric Mid‐Thigh Pull Protocols for Performance Diagnostics in Basketball Players

**DOI:** 10.1002/ejsc.70239

**Published:** 2026-08-18

**Authors:** Sumeng Xu, Hailong Yu, Tian Huang, Chuanzhi Wang, Longyan Yi, Bing Yan, Sulin Cheng, Xiuqiang Wang, Yang Wang, Olivier Girard

**Affiliations:** ^1^ China Institute of Sport and Health Science Beijing Sport University Beijing China; ^2^ Exercise Translational Medicine Center Institute of Translational Medicine Shanghai Jiao Tong University Shanghai China; ^3^ Sports Coaching College Beijing Sport University Beijing China; ^4^ Lab of Regenerative Medicine in Sports Science School of Physical Education and Sports Science South China Normal University Guangzhou China; ^5^ Key Laboratory for Performance Training and Recovery of General Administration of Sport Beijing Sport University Beijing China; ^6^ Center for Future Health and Intelligent Exercise Global Institute of Future Technology Shanghai Jiao Tong University Shanghai China; ^7^ School of Human Sciences (Exercise and Sport Science) The University of Western Australia Perth Australia

**Keywords:** characteristics, force–time, performance monitoring, rate of force development, sprint acceleration, strength diagnostics

## Abstract

To compare kinetic characteristics of a 1‐s isometric mid‐thigh pull (IMTP) protocol with the traditional 5‐s protocol and examine their associations with basketball‐specific performance. Twenty‐five Tier 3 male basketball players performed standardized 1‐s and 5‐s IMTP trials. Performance assessments included 20‐m linear sprint times (0–5, 0–10, and 0–20 m splits), countermovement jump and squat jump height, 505 and t‐test times. The 1‐s protocol produced slightly lower net absolute and relative peak force (*p* < 0.05, *g* = −0.28 to −0.25), but greater rate of force development (RFD) over 0–90, 0–200, and 0–250 ms (*p* < 0.05, *g* = 0.53 to 0.59). Absolute and relative peak force showed significant moderate‐to‐large correlations with 5‐m sprint time (*p* < 0.05, *r* = −0.41 to −0.52), whereas 1‐s RFD over 0–200 and 0–250 ms showed significant moderate‐to‐large correlations, with RFD over 0–200 ms also showing a significant moderate correlation with 20‐m time (*p* < 0.05, *r* = −0.40 to −0.51). Only 5‐s relative peak force showed a significant moderate correlation with 5‐m time (*p* < 0.05, *r* = −0.40). Squat jump height showed significant moderate‐to‐large correlations with peak force and RFD variables in both protocols (*p* < 0.05, *r* = 0.47 to 0.58), whereas countermovement jump height (*p* > 0.05, *r* = 0.13 to 0.39) and change‐of‐direction performance (*p* > 0.05, *r* = −0.27 to 0.01) showed no significant associations. The 1‐s IMTP protocol produced greater RFD despite slightly lower net peak force and showed broader associations with sprint acceleration performance.

## Highlights

- A shortened 1‐s IMTP protocol elicited greater rapid force production, despite producing slightly lower net peak force than the traditional 5‐s protocol.
- The 1‐s IMTP protocol showed broader associations with sprint acceleration performance, particularly across 5‐m, 10‐m, and 20‐m sprint times.
- Both 1‐s and 5‐s IMTP protocols were associated with squat jump height.

## 1 Introduction

Basketball is a high‐intensity intermittent sport that requires frequent accelerations, decelerations, changes of direction (COD), and repeated jumping and sprinting actions (Cao et al. 2025). Players perform approximately 1050 movement actions per game, including about ∼55 sprints and ∼44 jumps, with activity changing every ∼2 s (Conte et al. 2015). These actions are brief, typically lasting less than 3 s (Conte et al. 2015), whereas sprint efforts are predominantly short, with ∼57% covering 1–5 m and a further 30% covering 6–10 m (Ben Abdelkrim et al. 2007). These demands require athletes to rapidly produce high levels of force. From a force–time perspective, sprinting and jumping performance depend on both the magnitude of net propulsive force relative to body mass and the duration over which it is applied (Maffiuletti et al. 2016; Suchomel et al. 2016). Mass‐specific net force determines acceleration, whereas the force application over time determines mass‐specific net impulse and the resulting change in velocity, such as take‐off velocity during a countermovement jump. Consequently, maximal force capacity and rapid force production are complementary qualities that determine how much impulse can be generated within the time available for movement (Maffiuletti et al. 2016; Suchomel et al. 2016). Accurate assessment of an athlete's ability to rapidly produce force is therefore essential for performance profiling and training prescription (Häkkinen 1993; Bosco et al. 1983).

The isometric mid‐thigh pull (IMTP) is a safe and reliable method to quantify maximal and rapid force production characteristics and monitor neuromuscular fatigue under controlled conditions (Stone et al. 2019; Grgic et al. 2022; Drake et al. 2019; Comfort et al. 2019; Yang et al. 2025). Seminal work by Haff et al. (1997) established the foundation for IMTP research by characterizing force–time variables during dynamic and isometric mid‐thigh pull actions, including peak force (PF) and maximal rate of force development (RFD) derived from the greatest force–time slope. Subsequent methodological work has emphasized the importance of clearly defining IMTP force–time variables, as PF may be expressed as gross or net force, whereas RFD can be quantified as peak RFD, average RFD, and RFD calculated over predetermined time intervals (Comfort et al. 2019; Haff et al. 2015). These methodological differences influence both the magnitude and reliability of RFD measurements, with time‐band RFD generally demonstrating superior reliability, average RFD showing poorer reliability, and peak RFD being sensitive to the sampling window (Comfort et al. 2019; Haff et al. 2015). Accordingly, IMTP force–time variables have been shown to correlate with key physical performance indicators, such as sprint speed and jump height, across a range of athletic populations including team‐sport and basketball players (Wang et al. 2016; Scanlan et al. 2020; Townsend et al. 2019; Hogarth et al. 2021).

Traditionally, IMTP protocols require athletes to maintain a maximal contraction for ∼3–6 s to ensure attainment of true maximal force (Comfort et al. 2019; Wang et al. 2016; Scanlan et al. 2020; Townsend et al. 2019; Spiteri et al. 2014). Although effective for assessing maximal strength, this approach presents significant practical and methodological challenges in team‐sport settings. Administering multiple traditional IMTP trials across an entire squad can be time‐prohibitive, as the longer contraction duration and intertrial recovery periods cumulatively increase the overall testing time, thereby reducing the feasibility of routine monitoring (Suarez et al. 2022). More importantly, trial duration and testing intent may influence both the magnitude and reliability of force–time variables. Previous researchers have demonstrated that traditional longer IMTP protocols are better suited for assessing PF, whereas shortened or explosive protocols tend to elicit greater early force–time characteristics and may provide more reliable RFD measures (Drake et al. 2019; Guppy et al. 2022; Stevens et al. 2025; Suarez et al. 2022). Furthermore, longer contractions may promote subconscious pacing or a slower rate of force rise, as athletes prioritize force maintenance over explosive force expression (Drake et al. 2019; Stevens et al. 2025; Suarez et al. 2022).

These limitations are particularly relevant in basketball, where force must be applied within brief time frames and on‐court performance relies on rapid, ballistic force expression rather than sustained force output (Ziv and Lidor 2009). Accordingly, shortened, time‐constrained isometric protocols have been proposed as a more ecologically valid assessment approach. Drake et al. (2019) first evaluated this concept using a 1‐s isometric squat test (ISqT), demonstrating improved reliability and greater RFD values, although PF was reduced relative to traditional protocols. Suarez et al. (2022) found that a 1‐s IMTP elicited comparable PF while producing significantly higher RFD and impulse in weightlifters and powerlifters. Guppy et al. (2022) reported that a short‐duration IMTP protocol produced highly reliable RFD measures and greater early force–time characteristics, but lower PF than a traditional 5‐s protocol. They also noted that the reliability of time‐dependent force–time variables was affected by trial‐selection criteria. Consistent with these observations, Stevens et al. (2025) subsequently reported a ∼9% reduction in PF during shortened IMTP trials in elite athletes. Collectively, available evidence suggests that shortened protocols may enhance the assessment of RFD, but uncertainty remains regarding their ability to accurately capture maximal force capacity.

The discrepancies observed across studies may reflect methodological differences, including testing modalities (e.g., isometric squat (Drake et al. 2019) versus IMTP (Guppy et al. 2022; Stevens et al. 2025; Suarez et al. 2022)), participant characteristics (strength‐trained individuals (Drake et al. 2019; Guppy et al. 2022), weightlifters and powerlifters (Suarez et al. 2022), and elite mixed‐sport athletes (Stevens et al. 2025)), and verbal instructions (e.g., “explosive” vs. “maximal” cues (Drake et al. 2019; Guppy et al. 2022)). Consequently, it remains unknown whether a shortened 1‐s IMTP protocol performed under standardized instructions permits full expression of maximal force or systematically underestimates PF in basketball players. This question may be population‐specific, as differences in strength profiles and sport‐specific force demands can influence force–time characteristics (Suchomel et al. 2016). Importantly, previous investigations of shortened protocols have primarily examined reliability and differences in force–time outcomes (Drake et al. 2019; Guppy et al. 2022; Stevens et al. 2025; Suarez et al. 2022), with limited attention to their associations with sport‐specific performance. It therefore remains unclear whether kinetic variables derived from a 1‐s IMTP demonstrate stronger associations with basketball‐specific performance outcomes (i.e., sprint acceleration, vertical jumping, and COD) than those observed from traditional protocols. Clarifying this relationship has important practical implications for neuromuscular monitoring in basketball.

Therefore, we compared kinetic outputs derived from a 1‐s IMTP protocol with those obtained from a traditional 5‐s protocol in basketball players, using standardized verbal instructions to isolate the effect of temporal constraint. Additionally, we examined the associations between kinetic variables from both protocols and basketball‐specific dynamic performance. It was hypothesized that the 1‐s protocol would yield comparable PF and superior RFD values to the 5‐s condition and demonstrate stronger correlations with dynamic performance outcomes.

## 2 Methods

### 2.1 Subjects

An a priori sample size estimation was conducted using G*Power (version 3.1.9.2; G*Power, University of Düsseldorf, Düsseldorf, Germany). Based on moderate‐to‐large correlations previously reported between IMTP variables and sprint and jump performance (Scanlan et al. 2020; Townsend et al. 2019), an expected correlation coefficient of *r* = 0.55 was selected for a two‐tailed bivariate correlation analysis. With *α* = 0.05 and statistical power set at 0.80, the minimum required sample size was 23 participants. Twenty‐five Tier 3 (McKay et al. 2022) male basketball players (age 20.3 ± 2.2 y; height 187.0 ± 5.9 cm; body mass 78.1 ± 6.6 kg) were recruited for this study. All participants were free from known medical conditions and had no history of orthopedic surgery. Individuals reporting any musculoskeletal injury within the past 6 months were excluded. Before participation, all procedures, potential risks, and benefits were explained, and written informed consent was obtained. The study was approved by the local ethical committees and complied with the principles of the Declaration of Helsinki.

### 2.2 Experimental Design

This study employed a cross‐sectional design to compare kinetic outputs derived from two IMTP protocols (1‐s *vs*. 5‐s) and to examine their associations with basketball‐specific physical performance measures, including linear sprinting, vertical jumping, and COD ability. Participants completed four sessions, including one familiarization session and three testing sessions, each separated by at least 48 h. During familiarization, anthropometric measurements (body mass and height) were collected, followed by standardized instruction and supervised practice of all test procedures. Participants repeated each movement until correct execution was confirmed by the research team. The first test session involved IMTP assessment, during which participants performed both 1‐s and 5‐s IMTP trials in a standardized order after a standardized warm‐up. The second session assessed jumping performance (countermovement jump[CMJ] and squat jump [SJ]), whereas the third session evaluated speed and COD performance (20‐m sprint, t‐test, and 505 test). Standardized rest intervals were applied across all tests. All assessments were performed on the same indoor hardwood basketball court to control for surface variability. Participants were instructed to avoid strength training, vigorous exercise, or competition for 7 days before testing and to abstain from alcohol and caffeine for 24 h prior to each session.

### 2.3 Procedures

#### 2.3.1 Isometric Mid‐Thigh Pull

The IMTP was performed using a custom‐built rack with adjustable bar height to replicate the second pull position of the clean (Wang et al. 2016; Suarez et al. 2022). Participants adopted a self‐selected pulling posture, adjusting hip and knee angles to their preferred position (Wang et al. 2016; Suarez et al. 2022). Joint angles were measured using a goniometer, with knee and hip angles of 135.4 ± 6° and 145.6 ± 3°, respectively. These values were within the recommended ranges reported in a previous review (Comfort et al. 2019). Bar height, grip position, and joint angles were recorded during familiarization and replicated during testing to ensure consistency. Weightlifting straps were used to minimize the influence of grip strength on force production.

After a standardized warm‐up, participants completed two submaximal IMTP attempts at ∼50% and 75% of perceived maximal effort. After a 2‐min rest period, formal testing began. For both the 1‐s and 5‐s IMTP protocols, participants were instructed to apply a small and steady pretension against the bar before the initiation cue and then to “pull as fast and as hard as possible” until receiving the stop signal. A verbal countdown (“3–2–1”) preceded each attempt, followed by a loud “pull” command, with strong verbal encouragement provided throughout. Trial duration was controlled by the investigator using a stopwatch, with a verbal stop command given at 1 s for the 1‐s protocol and at 5 s for the 5‐s protocol. Each protocol consisted of three maximal trials. Trials displaying clear countermovement were discarded and repeated after adequate rest. Rest intervals were standardized at 30 s between trials for the 1‐s protocol and 120 s for the 5‐s protocol, with a 3‐min rest separating protocols (Suarez et al. 2022). To minimize potential fatigue associated with prolonged maximal contractions, the 1‐s protocol was completed before the 5‐s protocol (Suarez et al. 2022).

The rack was situated over two force plates (Kistler type 9286BA, Winterthur, Switzerland). Vertical ground reaction forces were sampled at 1000 Hz and recorded using BioWare software (Version 5.1; Kistler Instrument Corporation, Switzerland). Force onset was manually selected by the same experienced investigator across all trials (Suarez et al. 2022). Net absolute PF (N) was defined as the highest vertical ground reaction force achieved during each IMTP trial after subtracting body weight, whereas net relative PF (N·kg^−1^) was calculated by dividing net absolute PF by body mass. RFD was calculated as ΔForce/ΔTime over predetermined time intervals (0–90, 0–200, and 0–250 ms). These epochs were selected to represent the early (< 100 ms) and later (> 100 ms) phases of rapid force production, consistent with previous IMTP studies and methodological recommendations (Comfort et al. 2019; Guppy et al. 2022; Suarez et al. 2022). The 0–200 and 0–250 ms epochs were also considered relevant to the brief force‐application time frames required during sprinting, jumping, and change‐of‐direction tasks (Townsend et al. 2019). Peak RFD (pRFD) was defined as the maximum slope of the force–time curve within a 20‐ms moving window (Bosco et al. 1983; Stone et al. 2019; Grgic et al. 2022). For all variables, the highest value obtained across the three trials was retained for statistical analysis.

#### 2.3.2 Vertical Jumps

Vertical jump performance was assessed using CMJ and SJ. All testing was preceded by a standardized dynamic warm‐up and two submaximal practice attempts. Both CMJ and SJ were performed on the same force plates and analyzed using the same software as for the IMTP assessments. Jump height was calculated from take‐off velocity, derived by integrating net vertical ground reaction force over time using the impulse‐momentum relationship (Eythorsdottir et al. 2024). For the CMJ, participants were instructed to jump as high as possible using a self‐selected countermovement depth, with hands placed on the hips to eliminate the influence of arm swing. Three maximal trials were completed with 1 minute of rest, and the highest jump height was retained for analysis. For the SJ, participants started from a static position at ∼90° of knee flexion, with hands placed on the hips, consistent with the CMJ protocol. They were required to hold this position for at least 2 s to eliminate the contribution of the stretch‐shortening cycle before performing a maximal vertical jump. Three trials were completed with 1 min of rest, and the best performance was retained for analysis.

#### 2.3.3 Linear Sprints

Linear sprint performance was assessed over 20 m with electronic timing gates (SmartSpeed, Fusion Sport, Australia). Timing gates were positioned at a height of 1.2 m (Scanlan et al. 2020), approximately at participants' hip height to ensure the timing beam was triggered primarily by the torso, thereby minimizing false triggers and improving measurement consistency. After a standardized dynamic warm‐up, participants initiated each sprint from a standing start, with the lead foot positioned 0.5 m behind the first timing gate to prevent premature triggering. Participants completed three maximal sprints, with 3 min of passive recovery between attempts. Split times at 5 m, 10 m, and 20 m were automatically recorded, and the fastest trial was retained for analysis.

#### 2.3.4 Change of Direction Ability

COD performance was assessed using the t‐test and the 505 COD test, administered using electronic timing gates. All testing was preceded by a standardized dynamic warm‐up and two submaximal practice attempts.

The 505 test assessed the ability to rapidly decelerate, execute a 180° turn, and reaccelerate. A single electronic timing gate was positioned 10 m from the start line. Participants began from a standing start and sprinted toward the gate, continued for an additional 5 m, and planted either the left or right foot on or beyond a marked turning line before performing a 180° turn and sprinting 5 m back through the timing gate to complete the trial. Each participant performed three trials with 3 minutes of rest between attempts. Trials were repeated if participants failed to plant on or beyond the turning line or used additional steps to complete the turn. The fastest time was recorded for analysis.

For the t‐test, electronic timing gates were positioned at the start and finish lines. Participants began from a standing start, with the lead foot positioned 0.5 m behind the timing gate to prevent premature triggering. Participants sprinted forward to the 5‐m cone and touched it with one hand, then shuffled laterally to the left cone and touched it, followed by a lateral shuffle to the right cone. Maintaining a forward‐facing orientation and without crossing their feet, participants then shuffled 2.5 m back to the central cone, touched it, and immediately backpedaled to the finish line, passing through the timing gate to complete the trial. Trials were deemed invalid and repeated if participants crossed their feet during lateral movement, failed to touch a cone, or did not maintain a forward‐facing orientation. Three trials were completed with 3 minutes of rest, and the fastest time was recorded for analysis.

### 2.4 Statistical Analyses

Analyses were performed using R software (version 4.4.1; R Foundation for Statistical Computing, Vienna, Austria). Data are presented as mean (SD). The Shapiro–Wilk test was used to assess normality of each variable. Test–retest reliability was determined by calculating the intraclass correlation coefficient (ICC), coefficient of variation (CV), and 95% CI. ICC values were interpreted using the lower bound of the 95% CI according to the following thresholds: *poor* (< 0.50), *moderate* (≥ 0.50 to < 0.75), *good* (≥ 0.75 to < 0.90), and *excellent* (≥ 0.90) (Koo and Li 2016). The coefficient of variation (CV) was calculated as the within‐subject SD divided by the mean and multiplied by 100 and interpreted as *excellent* (< 5%), *good* (≥ 5% to < 10%), *acceptable* (≥ 10% to ≤ 15%), and *unacceptable* (> 15%) (Shechtman 2013). Variables were considered to demonstrate acceptable reliability if the lower bound of the ICC 95% CI indicated at least *moderate* reliability and CV was ≤ 15%. Only IMTP‐derived variables that met the minimum acceptable reliability criteria were retained for subsequent correlation analyses with physical performance measures. Paired samples *t*‐tests were performed to compare force–time characteristics between the 1‐s and 5‐s protocols. To control for family‐wise error, *p* values were adjusted using the Holm–Bonferroni correction (Holm 1979). The magnitude of differences was assessed using Hedges' g with 95% confidence intervals (CIs). The absolute magnitude of Hedges' g was interpreted as *trivial* (< 0.20), *small* (≥ 0.20 to < 0.60), *moderate* (≥ 0.60 to < 1.20), *large* (≥ 1.20 to < 2.00), and *very large* (≥ 2.00) (Hopkins et al. 2009). Relationships between IMTP variables and physical performance measures were assessed using Pearson's correlation coefficient (*r*). Correlation magnitudes were interpreted using absolute values as *trivial* (< 0.10), *small* (≥ 0.10 to < 0.30), *moderate* (≥ 0.30 to < 0.50), *large* (≥ 0.50 to < 0.70), *very large* (≥ 0.70 to < 0.90), and *almost perfect* (≥ 0.90). Statistical significance was set at *p* ≤ 0.05.

## 3 Results

Within‐session test–retest reliability statistics for all kinetic variables are presented in Table 1. Based on the lower bound of the 95% CI, absolute and relative PF demonstrated *excellent* ICC reliability in the 1‐s protocol and *good* ICC reliability in the 5‐s protocol, with excellent CVs across both protocols (ICC = 0.92–0.95; 95% CI lower bound = 0.85–0.92; CV = 3.1%–3.8%). Late‐phase RFD variables (RFD_0–200 ms_ and RFD_0–250 ms_) showed *moderate‐to‐good* ICC reliability, with *good‐to‐acceptable* CVs (ICC = 0.75–0.87; 95% CI lower bound = 0.58–0.76; CV = 7.2%–12.1%). Peak RFD showed poor‐to‐moderate ICC reliability, with *acceptable‐to‐unacceptable* CVs (ICC = 0.61–0.71; 95% CI lower bound = 0.35–0.51; CV = 13.6%–15.5%). RFD_0–90 ms_ demonstrated poor ICC reliability in both protocols, with *unacceptable* CVs (ICC = 0.57–0.59; 95% CI lower bound = 0.35–0.36; CV = 18.2%–19.4%).

**TABLE 1:** Within‐session test–retest reliability of isometric mid‐thigh pull kinetic variables.

Variables	1‐s Protocol		5‐s Protocol	
	ICC (95% CI)	CV% (95% CI)	ICC (95% CI)	CV% (95% CI)
Absolute PF	0.95 (0.90–0.97)	3.1 (2.4–3.8)	0.93 (0.85–0.94)	3.8 (2.7–4.9)
Relative PF	0.95 (0.92–0.98)	3.1 (2.4–3.8)	0.92 (0.85–0.96)	3.8 (2.7–4.9)
Peak RFD	0.61 (0.35–0.77)	15.5 (12.2–18.8)	0.71 (0.51–0.84)	13.6 (9.6–17.6)
RFD_0–90 ms_	0.57 (0.35–0.76)	18.2 (15.0–21.5)	0.59 (0.36–0.77)	19.4 (14.1–24.6)
RFD_0–200 ms_	0.84 (0.71–0.92)	7.6 (5.6–9.7)	0.75 (0.58–0.87)	12.1 (8.2–16.0)
RFD_0–250 ms_	0.87 (0.76–0.93)	7.2 (5.4–9.0)	0.81 (0.66–0.90)	10.7 (7.3–14.1)

Abbreviations: CI, confidence interval; CV, coefficient of variation; ICC, intraclass correlation coefficient; PF, peak force; RFD, rate of force development.

**FIGURE 1:**
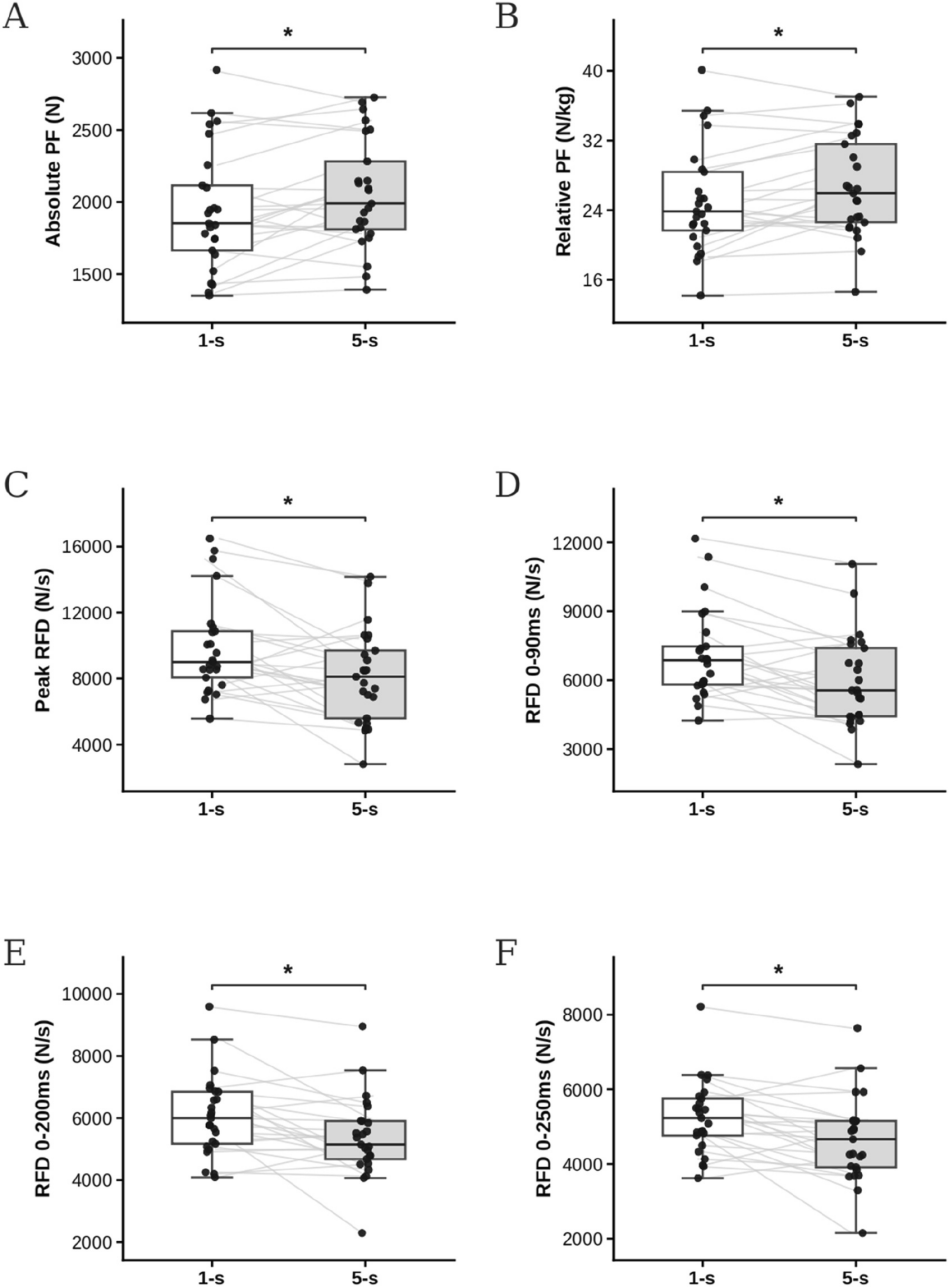
Comparison of absolute and relative peak force (A, B) and rate of force development (C–F) between the 1‐s and 5‐s isometric mid‐thigh pull (IMTP) protocols. Box‐and‐whisker plots represent the median (central line), interquartile range (box), and minimum and maximum values (whiskers). Individual data points are displayed, with gray lines connecting the same participant across conditions to visualize individual responses. PF, peak force; RFD, rate of force development. *p < 0.05.

Differences in IMTP kinetic metrics between the 1‐s and 5‐s protocols are presented in Table 2 and illustrated in Figure 1. Paired‐samples *t*‐tests with Holm–Bonferroni correction showed that the 1‐s protocol produced significantly lower absolute PF and relative PF than the 5‐s protocol (*p* = 0.044), with *small* effect sizes (*g* = −0.28 and −0.25, respectively) (Figure 1A‐B). In contrast, the 1‐s protocol yielded significantly higher values for all RFD metrics (RFD_0–90 ms,_ RFD_0–200 ms_, RFD_0–250 ms_, and pRFD) compared to the 5‐s protocol (*p* < 0.05) (Figure 1C‐F), with *small* effect sizes (*g* = 0.53–0.59).

**TABLE 2:** Comparison of kinetic variables between 1‐s and 5‐s protocols (Mean ± SD).

Variables	1‐s Protocol	5‐s Protocol	*p* value	Hedges' *g* (95% CI)
Absolute PF, N	1942 ± 422	2057 ± 379	0.044*	−0.28 (−0.52 to −0.04)
Relative PF, N/kg	25.1 ± 6.1	26.6 ± 5.7	0.044*	−0.25 (−0.47 to −0.04)
Peak RFD, N/s	9853 ± 2884	8125 ± 2810	0.014*	0.59 (0.20–0.97)
RFD_0–90 ms_, N/s	7075 ± 1954	5946 ± 1988	0.006*	0.55 (0.22–0.89)
RFD_0–200 ms_, N/s	6074 ± 1299	5363 ± 1279	0.016*	0.53 (0.16–0.91)
RFD_0–250 ms_, N/s	5248 ± 986	4647 ± 1127	0.014*	0.56 (0.19–0.91)

Abbreviations: CI, confidence interval; PF, peak force; RFD, rate of force development.

^*^ Significant difference (*p* < 0.05).

**FIGURE 2:**
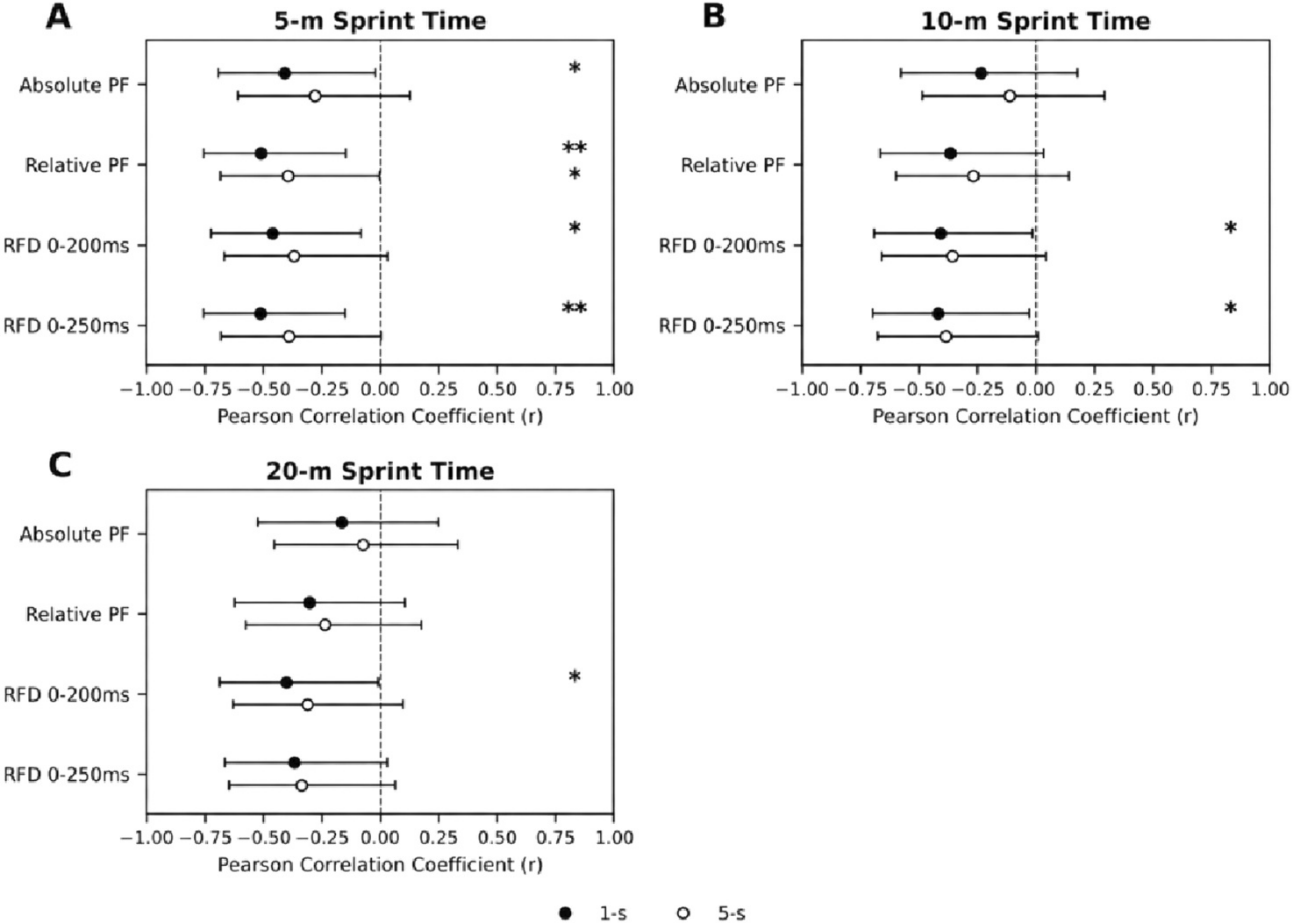
Pearson correlation coefficients (*r*) with 95% confidence intervals describing the relationships between isometric mid‐thigh pull (IMTP) kinetic metrics and linear sprint performance at 5‐m (A), 10‐m (B), and 20‐m (C) intervals. Data are shown for both the 1‐s and 5‐s (black and white circles, respectively) IMTP protocols. *p < 0.05; **p < 0.01.

**FIGURE 3:**
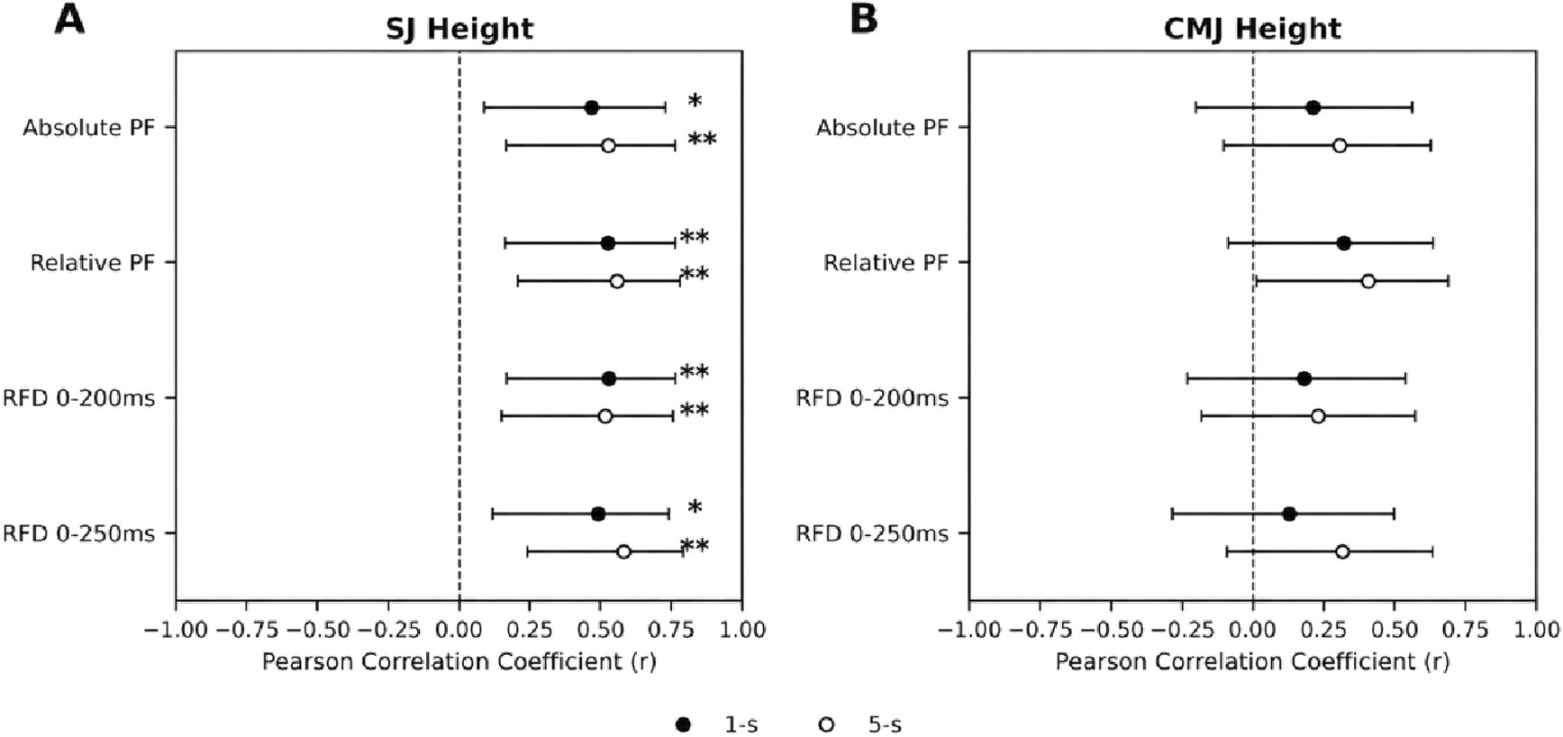
Pearson correlation coefficients (*r*) with 95% confidence intervals describing the relationships between isometric mid‐thigh pull (IMTP) kinetic metrics and vertical jump height in the squat jump (SJ, A) and countermovement jump (CMJ, B). Data are shown for both the 1‐s and 5‐s (black and white circles, respectively) IMTP protocols. *p < 0.05; **p < 0.01.

**FIGURE 4:**
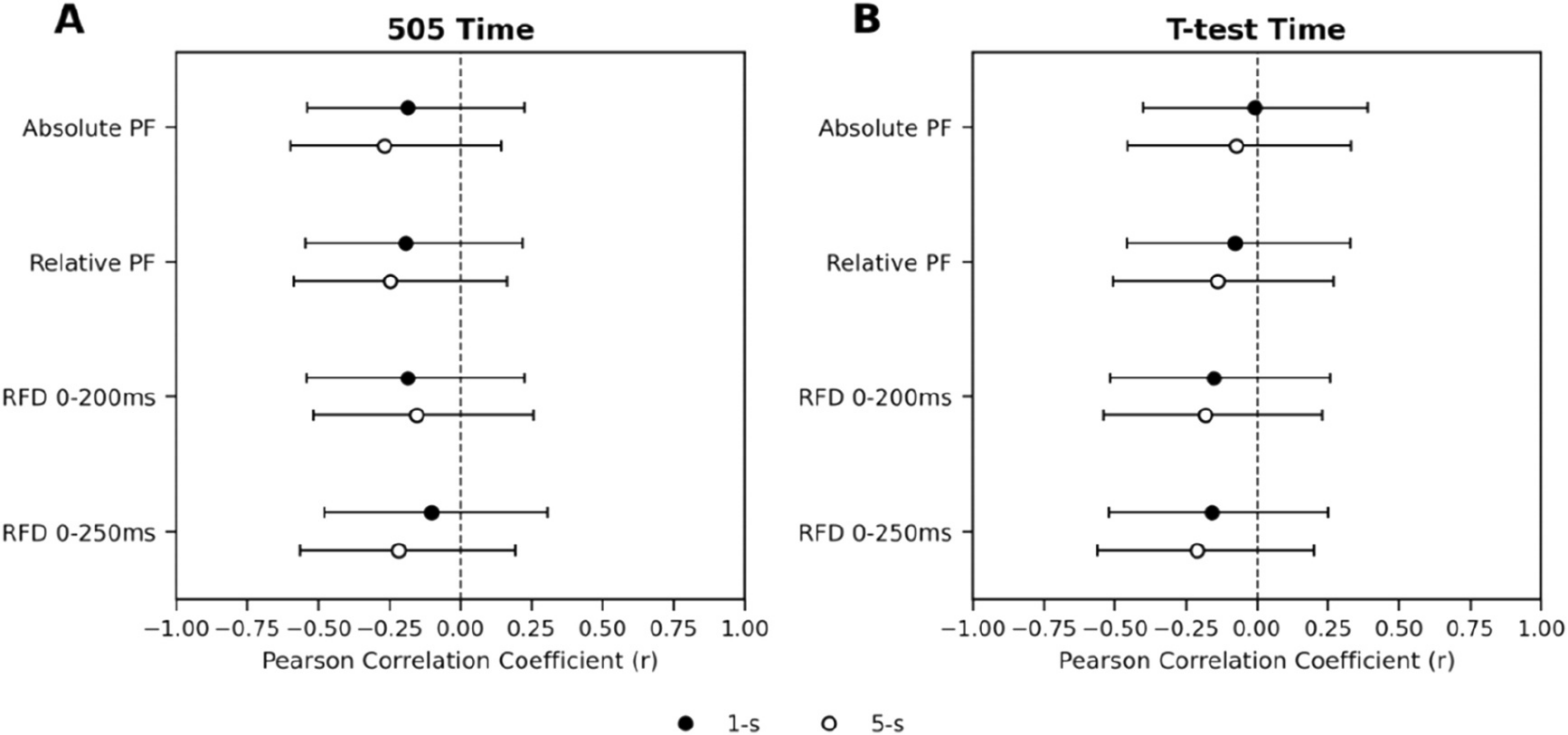
Pearson correlation coefficients (*r*) with 95% confidence intervals describing the relationships between isometric mid‐thigh pull (IMTP) kinetic metrics and change‐of‐direction performance in the 505 test (A) and t‐test (B). Data are shown for both the 1‐s and 5‐s (black and white circles, respectively) IMTP protocols. *p < 0.05; **p < 0.01.

Pearson correlation coefficients describing relationships between IMTP kinetic variables and dynamic performance measures are presented in Table 3. *Moderate*‐to*‐large* correlations were observed between 5‐m sprint time and all variables derived from the 1‐s protocol (Figure 2A), including absolute PF (*r* = −0.41, *p* < 0.05), relative PF (*r* = −0.52, *p* < 0.01), RFD_0–200 ms_ (*r* = −0.46, *p* < 0.05), and RFD_0–250 ms_ (*r* = −0.51, *p* < 0.01). Although relative PF from the 5‐s protocol was also significantly associated with 5‐m sprint time (*r* = −0.40, *p* < 0.05), the magnitude of this association was smaller than that observed for the 1‐s condition. Furthermore, no significant correlations were found between 5‐m sprint time and any 5‐s RFD metrics (*p* > 0.05). Associations between IMTP variables and sprint performance decreased with increasing sprint distance; however, 1‐s RFD metrics remained moderately associated with 10‐m and 20‐m times (*r* = −0.40 to −0.42, *p* < 0.05; Figure 2B‐C), whereas no significant associations were found for the 5‐s protocol. PF metrics from both protocols demonstrated *moderat* *e*‐to‐*large* associations with SJ height (*r* = 0.47 to 0.55, *p* < 0.05; Figure 3A), with stronger relationships evident for relative PF than absolute PF. For RFD metrics, late‐phase RFD variables showed *moderate to large* correlations with SJ height (*r* = 0.49 to 0.58, *p* < 0.05). Specifically, RFD_0–200 ms_ demonstrated large correlations in both the 1‐s and 5‐s protocols, whereas RFD_0–250 ms_ showed a moderate correlation in the 1‐s protocol and a large correlation in the 5‐s protocol. No significant correlations were observed between any IMTP metrics and CMJ height (Figure 3B). Likewise, none of the retained IMTP variables were significantly associated with either 505 test time (Figure 4A) or t‐test time (Figure 4B).

**TABLE 3:** Pearson correlation coefficients (*r*) and 95% confidence intervals between isometric mid‐thigh pull kinetic variables (1‐s and 5‐s protocols) and dynamic performance measures in basketball players.

	Linear sprint			Vertical jump		Change of direction	
	5‐m sprint time	10‐m sprint time	20‐m sprint time	SJ height	CMJ height	505 time	*t*‐test time
Absolute PF							
1‐s protocol	*r* = −0.41* (−0.69 to −0.02)	*r* = −0.24 (−0.58 to 0.18)	*r* = −0.16 (−0.53 to 0.25)	*r* = 0.47* (0.09–0.73)	*r* = 0.21 (−0.20 to 0.56)	*r* = −0.18 (−0.54 to 0.23)	*r* = 0.01 (−0.40 to 0.39)
5‐s protocol	*r* = −0.28 (−0.61 to 0.13)	*r* = −0.11 (−0.49 to 0.29)	*r* = −0.07 (−0.46 to 0.33)	*r* = 0.53** (0.17–0.76)	*r* = 0.31 (−0.10 to 0.63)	*r* = −0.27 (−0.60 to 0.14)	*r* = −0.07 (−0.45 to 0.33)
Relative PF							
1‐s protocol	*r* = −0.52** (−0.75 to −0.15)	*r* = −0.39 (−0.67 to 0.03)	*r* = −0.30 (−0.62 to 0.10)	*r* = 0.52** (0.16–0.76)	*r* = 0.31 (−0.10 to 0.63)	*r* = −0.19 (−0.55 to 0.22)	*r* = −0.08 (−0.46 to 0.33)
5‐s protocol	*r* = −0.40* (−0.68 to −0.01)	*r* = −0.29 (−0.60 to 0.14)	*r* = −0.24 (−0.58 to 0.17)	*r* = 0.55** (0.20–0.78)	*r* = 0.39 (−0.01 to 0.68)	*r* = −0.25 (−0.58 to 0.16)	*r* = −0.14 (−0.51 to 0.27)
RFD_0–200 ms_							
1‐s protocol	*r* = −0.46* (−0.72 to −0.08)	*r* = −0.40* (−0.69 to −0.01)	*r* = −0.40* (−0.69 to −0.01)	*r* = 0.53** (0.17–0.76)	*r* = 0.18 (−0.23 to 0.54)	*r* = −0.19 (−0.54 to 0.23)	*r* = −0.15 (−0.52 to 0.26)
5‐s protocol	*r* = −0.36 (−0.67 to 0.03)	*r* = −0.35 (−0.66 to 0.04)	*r* = −0.31 (−0.63 to 0.09)	*r* = 0.52** (0.15–0.76)	*r* = 0.23 (−0.18 to 0.57)	*r* = −0.16 (−0.52 to 0.26)	*r* = −0.18 (−0.54 to 0.23)
RFD_0–250 ms_							
1‐s protocol	*r* = −0.51** (−0.76 to −0.15)	*r* = −0.42* (−0.70 to −0.03)	*r* = −0.37 (−0.67 to 0.03)	*r* = 0.49* (0.12–0.74)	*r* = 0.13 (−0.28 to 0.50)	*r* = −0.11 (−0.48 to 0.31)	*r* = −0.16 (−0.52 to 0.25)
5‐s protocol	*r* = −0.38 (−0.68 to 0.01)	*r* = −0.38 (−0.68 to 0.01)	*r* = −0.34 (−0.65 to 0.06)	*r* = 0.58** (0.24–0.79)	*r* = 0.32 (−0.09 to 0.63)	*r* = −0.22 (−0.56 to 0.20)	*r* = −0.21 (−0.56 to 0.20)

Abbreviations: PF, peak force; RFD, rate of force development.

^*^ *p* < 0.05.

^**^ *p* < 0.01.

## 4 Discussion

The 1‐s IMTP protocol elicited significantly higher early phase RFD but slightly lower absolute and relative PF than the traditional 5‐s protocol. The enhanced rapid force characteristics observed under the shortened protocol broadly align with previous findings (Guppy et al. 2022; Stevens et al. 2025; Suarez et al. 2022). Although verbal instructions were standardized, the imposed temporal constraint likely acted as an intrinsic stimulus (Suarez et al. 2022), minimizing subconscious pacing and facilitating faster force expression. The lower PF observed in the 1‐s protocol is consistent with Guppy et al. (2022) and Stevens et al. (2025), but contrasts with Suarez et al. (2022), who reported comparable PF between shortened and traditional protocols. This discrepancy may reflect differences in participant characteristics. Previous researchers have examined a mixed‐sex cohort across nine different sports (Stevens et al. 2025), strength‐trained individuals (Guppy et al. 2022), and weightlifters and powerlifters (Suarez et al. 2022). The strength‐specialized athletes studied by (Suarez et al. 2022) likely possessed neuromuscular adaptations that enabled them to attain PF more rapidly. In contrast, our sample consisted exclusively of male basketball players. Despite their frequent exposure to rapid force production and short ground‐contact times (Spiteri et al. 2014; Stojanović et al. 2018), the 1‐s constraint still limited maximal force expression at the group level. Differences in relative strength between cohorts further support this interpretation. The PF values reported by Suarez et al. (2022), when normalized to body mass, correspond to approximate gross relative PF values of 48.3 and 49.5 N·kg^−1^ for the shortened and traditional protocols, respectively. In comparison, the present basketball players achieved substantially lower values (34.9 ± 6.0 and 36.4 ± 5.6 N·kg^−1^, respectively). Therefore, the lower relative force‐producing capacity of the present cohort may partly explain why the 1‐s constraint limited maximal PF expression, whereas the strength‐specialized athletes in the study by Suarez et al. (2022) achieved comparable PF regardless of protocol duration.

Although the 5‐s protocol produced slightly greater PF overall, individual responses were heterogeneous, consistent with the findings of Stevens et al. (2025). Notably, 9 of the 25 athletes produced equal or greater net PF during the 1‐s protocol. This variability suggests that the influence of protocol duration likely depends on individual force–time strategies or neuromuscular characteristics rather than representing a consistent underestimation of PF. Athletes capable of attaining maximal PF within 1 s may possess a distinct neuromuscular profile characterized by greater rapid force accessibility, which may also underpin superior explosive performance. Future studies should investigate the characteristics of these athletes to determine whether shortened IMTP protocols provide complementary information beyond traditional maximal strength assessment. Although the 1‐s protocol enhanced rapid force expression, the traditional 5‐s IMTP remains the preferred option when the primary objective is to obtain the highest estimate of maximal isometric strength while minimizing the likelihood of underestimating PF across individuals.

Another novel finding is that the 1‐s protocol showed broader associations with sprint acceleration performance than the traditional 5‐s protocol. Specifically, 1‐s relative PF (*r* = −0.52) and 1‐s RFD_0–250 ms_ (*r* = −0.51) emerged as the strongest kinetic correlates of 5‐m sprint performance. By comparison, the correlation between relative PF derived from the 5‐s protocol and 5‐m sprint time (*r* = −0.40) closely aligns with values reported by Scanlan et al. (2020) (*r* = −0.44), confirming the *moderate* relationship typically observed between conventional IMTP protocols and initial acceleration in basketball players. Importantly, the broader association pattern observed for the 1‐s protocol suggests that it captures force–time characteristics more relevant to sprint acceleration performance. Initial sprint acceleration requires overcoming inertia, for which mass‐specific force production is a primary mechanical determinant (Weyand et al. 2010). Despite producing slightly lower net relative PF, the 1‐s protocol demonstrated a stronger association with 5‐m sprint performance, suggesting that it may better reflect force production under time‐constrained conditions. The 5‐s protocol permits athletes to achieve maximal force irrespective of how rapidly force is developed. In contrast, the 1‐s protocol acts as a temporal constraint that necessitates ballistic intent (Maffiuletti et al. 2016; Sahaly et al. 2001), effectively emphasizing early phase force expression within the brief ground contact times characteristic of sprint acceleration (Andersen and Aagaard 2006). Consequently, 1‐s relative PF reflects not only force capacity but also the ability to rapidly access that force. This interpretation is further supported by the significantly greater RFD values observed under the 1‐s protocol. Accordingly, the traditional 5‐s IMTP may underestimate explosive force expression by permitting a more gradual force rise. In contrast, the elevated RFD outputs in the 1‐s protocol better reflect the force–time demands of sprint acceleration, thereby providing a plausible explanation for the stronger correlations observed. Consistent with this interpretation, moderate associations were also observed between 1‐s RFD variables and 10‐ and 20‐m sprint performance (*r* = −0.40 to −0.42), suggesting that the 1‐s protocol may provide performance‐relevant information beyond the initial 5 m of sprinting.

For SJ performance, absolute PF, relative PF, RFD_0–200 ms_, and RFD_0–250 ms_ from both protocols were significantly associated with SJ height; however, these associations were marginally stronger for the 5‐s protocol (*r* = 0.52 to 0.58) than for the 1‐s protocol (*r* = 0.47 to 0.53), particularly for RFD_0–250 ms_ (5‐s: *r* = 0.58; 1‐s: *r* = 0.49). Because PF was slightly greater in the 5‐s protocol, its marginally stronger associations may partly reflect the greater opportunity to attain maximal force during the longer contraction. Although the 1‐s protocol emphasizes rapid force production, the 5‐s protocol permits greater PF expression and a more stable force plateau. Because SJ height is mechanically determined by net vertical impulse during the concentric phase, performance depends not solely on PF attainment but also on the ability to maintain force throughout propulsion (Kirby et al. 2011; Thomas et al. 2015). Consequently, the traditional 5‐s protocol may better reflect this force‐maintenance capacity, explaining descriptively stronger associations observed for some variables in the 5‐s condition. Importantly, these differences in correlation magnitude do not necessarily indicate that the 1‐s protocol is less suitable for assessing SJ‐related force‐production characteristics. Although slightly stronger associations were observed for the 5‐s protocol, the 1‐s protocol also demonstrated significant moderate‐to‐large relationships across the same variables. These findings suggest that the 1‐s protocol captures force–time characteristics relevant to SJ performance without requiring extended contractions. Thus, although the 5‐s protocol may provide marginally stronger descriptive associations with SJ height, the 1‐s protocol remains a time‐efficient alternative for assessing SJ‐related force‐production capacity in basketball players.

In contrast to the SJ findings, the 5‐s IMTP protocol failed to exhibit significant correlations with CMJ height, consistent with previous investigations (Scanlan et al. 2020; Thomas et al. 2015). The imposition of a 1‐s temporal constraint did not alter this outcome, as no significant associations were observed under the shortened condition. This lack of association is likely explained by the mechanical specificity of the respective tasks. Although SJ performance is primarily determined by concentric force production, CMJ performance depends heavily on stretch‐shortening cycle utilization and elastic energy storage (Komi 2000). Because the IMTP is a purely isometric assessment without an eccentric pre‐stretch, it does not capture the elastic and reflexive mechanisms underpinning CMJ performance. Consequently, manipulating IMTP duration alone (i.e., adopting a 1‐s protocol) was insufficient to overcome this fundamental mechanical mismatch, explaining the persistent lack of meaningful correlations observed.

After excluding variables with insufficient reliability, neither the 1‐s nor the 5‐s IMTP protocol showed meaningful associations with COD performance. This may reflect the multifactorial nature of COD ability, which depends not only on maximal and rapid vertical force production but also on deceleration capacity, eccentric strength, braking strategy, center‐of‐mass control, technical execution, and the effective application of horizontal and lateral ground reaction forces (Spiteri et al. 2014; Dos'Santos et al. 2017). As a uniplanar, static isometric assessment, the IMTP may not adequately capture these complex dynamic and multidirectional demands. Therefore, the absence of meaningful associations should not be interpreted as evidence that force‐production qualities are unimportant for COD performance. Rather, it suggests that the retained IMTP variables may have limited task specificity for assessing COD ability in basketball players. Future research incorporating reliable and more task‐specific dynamic assessments may help clarify the contribution of rapid force‐production qualities to COD performance.

Several limitations should be acknowledged. First, the cohort was analyzed as a single group without stratification by playing position (e.g., guards, forwards, and centers). Given the distinct anthropometric profiles and movement demands across positions (Spiteri et al. 2014; Ben Abdelkrim et al. 2010), position‐specific differences may exist. However, subgroup analyses were not feasible with the present sample size and would have resulted in inadequately small positional groups. Consequently, the present findings cannot determine whether the diagnostic utility of the 1‐s and 5‐s IMTP protocols differs by playing position. Future research should recruit larger cohorts to enable position‐specific analyses, as the diagnostic utility of the 1‐s and 5‐s IMTP protocols may differ by playing role. Second, the IMTP was performed bilaterally, whereas sprinting and COD tasks are inherently unilateral. This mismatch in base of support and force‐vector orientation may limit the transferability of bilateral isometric measures to dynamic tasks reliant on single‐leg force production. Future investigations should therefore consider unilateral assessments to enhance ecological validity and predictive sensitivity. Furthermore, it remains unclear whether the differences observed between the 1‐s and 5‐s protocols under bilateral conditions would persist in unilateral configurations, where greater instability may differentially affect rapid force expression under varying temporal constraints.

## 5 Conclusion

A 1‐s IMTP protocol is a practical, time‐efficient alternative to the traditional 5‐s protocol for assessing selected force–time characteristics in basketball players. Although the 1‐s protocol elicited slightly lower absolute and relative PF, it produced greater RFD and showed broader associations with sprint acceleration performance. Both protocols were associated with SJ height, although the 5‐s protocol showed slightly stronger relationships. These findings suggest that the 1‐s IMTP may be preferable when testing efficiency and rapid force expression are priorities, whereas the traditional 5‐s protocol remains the more appropriate option when maximal strength assessment is the primary objective.

## Author Contributions

Research concept and study design: Sumeng Xu, Hailong Yu, Yang Wang, Longyan Yi. literature review: Sumeng Xu, Hailong Yu, Chuanzhi Wang. data collection: Bing Yan, Tian Huang, Hailong Yu. data analysis and interpretation: Sumeng Xu, Yang Wang, Longyan Yi, Tian Huang. statistical analyses: Sumeng Xu, Chuanzhi Wang, Sulin Cheng, Xiuqiang Wang. writing of the manuscript: Sumeng Xu, Olivier Girard, Xiuqiang Wang. reviewing/editing a draft of the manuscript: Sumeng Xu, Olivier Girard, Bing Yan, Xiuqiang Wang, Sulin Cheng.

## Funding

The authors have nothing to report.

## Ethics Statement

All procedures performed were in accordance with the ethical standards outlined by the institutional research committees of Beijing Sport University (no. 2023201H) and in conformity with the 1964 Helsinki declaration and its later amendments.

## Consent

All participants provided written informed consent prior to participation.

## Conflicts of Interest

The authors declare no conflicts of interest.

## Data Availability

The data that support the findings of this study are available from the corresponding author upon reasonable request.
